# Changes in specific leaf area of dominant plants in temperate grasslands along a 2500-km transect in northern China

**DOI:** 10.1038/s41598-017-11133-z

**Published:** 2017-09-07

**Authors:** Mengzhou Liu, Zhengwen Wang, Shanshan Li, Xiaotao Lü, Xiaobo Wang, Xingguo Han

**Affiliations:** 1 Erguna Forest-Steppe Ecotone Research Station, Institute of Applied Ecology, Chinese Academy of Sciences, Shenyang, 110016 China; 2 University of Chinese Academy of Sciences, Beijing, 100049 China; 3 Key Laboratory for Vegetation Ecology, Ministry of Education, Northeast Normal University, Changchun, 130024 China; 4 State Key Laboratory of Vegetation and Environmental Change, Institute of Botany, Chinese Academy of Sciences, Beijing, 100093 China

## Abstract

Specific leaf area (SLA) is a key trait with great ecological importance as it correlates with whole plant growth. We aimed to investigate how SLA varies with environmental factors at a geographical scale in temperate grasslands. We measured SLA and mass-based leaf nitrogen content (*N*_mass_) of four dominant plant genera along a 2500 km climatic gradient in northern China grassland, and correlated SLA with mean annual precipitation (MAP), mean annual temperature (MAT), soil nitrogen concentration (soil N), soil C:N and *N*_mass_. Climate accounts much more for SLA variation than soil variables for *Stipa*, *Cleistogens* and *Carex*. SLA of *Stipa* is negatively associated with MAP and soil N, while positively with MAT, but *Cleistogenes* and *Carex* show the opposite. For *Leymus*, soil N promotes SLA and accounts for largest fraction of SLA variation. Overall, SLA was positively correlated with *N*_mass_ in semi-arid regions, but not significant in arid regions. The genus-dependent responses of SLA may have consequences on ecosystem functioning, thus may help to predict the community composition and ecosystem functions under future climate scenario. The finding of SLA-*N*_mass_ trade-off and its susceptibility to precipitation will advance our understanding on plant resource use strategies.

## Introduction

Plant leaves play an essential role in biogeochemical cycles in ecosystems and thus changes of leaf traits strongly affect plant growth and production^1–3^.The investigation of plant leaf traits and their responses to the environmental change has gained increasingly more attention among ecologists in recent decades^4–8^. Specific leaf area (SLA), defined as the ratio of total leaf area to total leaf dry mass^9, 10^, has been shown one of the leaf traits best reflecting whole plant growth^11, 12^. SLA plays an important role in linking plant carbon (C) and water cycles because it describes the distribution of leaf biomass relative to leaf area, and thus refers to carbon gain relative to water loss, within a plant canopy^10, 13^.

Ecologists have shown that SLA varies with environmental factors: positively with precipitation at regional and global scales^2, 14, 15^, as well as with soil fertility and nutrient availability^2, 16–19^, whereas negatively with soil C:N ratio^7^. Unlike the consistent positive correlation between SLA and precipitation, the relationship between SLA and air temperature seems quite controversial^20, 21^. In general, the responses of SLA to air temperature were species-specific^22^, suggesting that different species have different mechanisms to cope with temperature changes which are largely related to the plant’s structure as a whole^23^.

Besides being correlated with environmental variables, SLA was shown to vary closely with other leaf traits: positively with mass-based leaf nitrogen content (*N*_mass_) and negatively with leaf lifespan^1, 2, 19, 24, 25^. It has been well established that the positive correlation between SLA and *N*_mass_ reflects the trade-off between two opposing resource strategies, i.e., conservation vs. rapid acquisition of soil water and nutrients^2, 26^. However, it is important to point out that these results were all obtained in areas with relatively high annual rainfall values, or the datasets used in these studies included very few cases from dry biomes comparable to those from wet or moist biomes. The relationship between SLA and *N*_mass_ might be different along an extensive resource gradient (in water and/or nutrient availability). It is well established that plants growing in nutrient-rich environments generally grow leaves with high N contents and a relatively short lifespan producing large amount of nutrient-rich litter, whereas plants growing on dry soils conserve water and nutrients in long-lived and recalcitrant tissues^2, 4, 7, 24, 26^. Thus, along a long gradient in climate and corresponding soil characters, there might be a complex relationship between SLA and *N*_mass_ indicating a trade-off between growth rate and nutrient conservation for plants.

The effects of soil and climate on SLA and other leaf traits have been evaluated at a global scale^7, 8^, however, the strength of these syntheses comes from their use of extraordinarily rich datasets, thus suffer from the difficulty in assuring standard data collection^3^. Moreover, the global studies encompassing all kinds of biomes might have overlooked more subtle patterns, which might be discovered from single-biome-focused and thus more refined studies. Therefore, large-scale empirical investigations conducted following a same protocol and focusing on a single type of biome are badly needed. After all, it is important to investigate how a “general” relationship found at global scale applies to more specific systems, to improve our understanding of the possibility to downscale such general findings. Indeed, it has been reported that relationships in the grassland biome were significantly different from the global average^27^.

**Figure 1:**
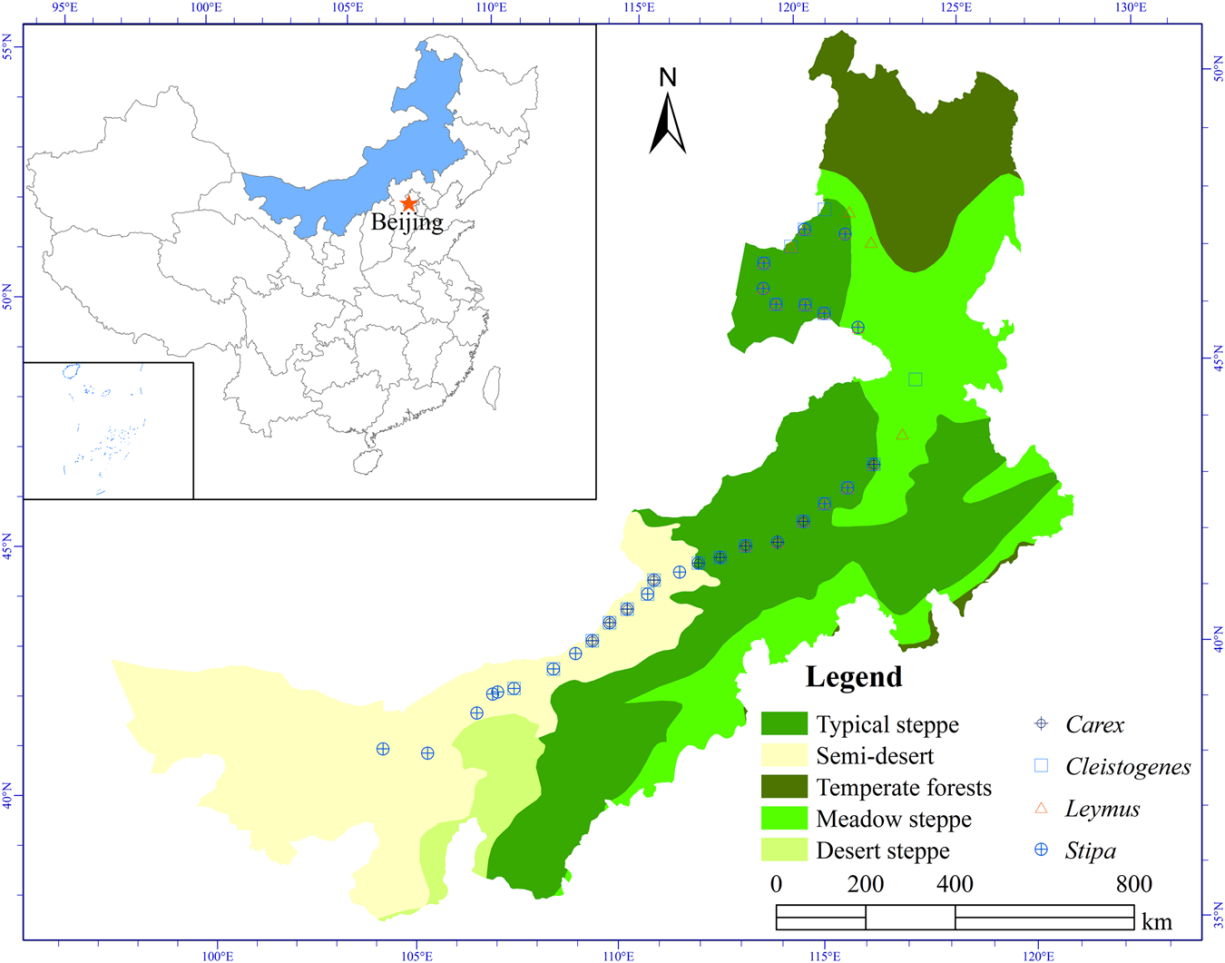
The transect and locations of sampling sites in the temperate grasslands of Northern China. The map was created using ArcGIS 9.3 (Esri, CA, http://www.esri.com).

Here, we sampled the leaves in 38 sites following the same protocol and investigated relationships between SLA and both climate factors and soil properties along a 2500 km long climatic gradient through the temperate grassland biome in northern China (Fig. 1; Table 1). Along the transect, the most dominant genera are *Stipa*, *Cleistogenes*, *Leymus* and *Carex*, with one to three species present for each genus (Table 2). We quantified the relationship of SLA of the four dominant genera with environmental variables along the transect. We addressed the following questions: (i) To what extent do mean annual precipitation (MAP), mean annual temperature (MAT), aridity index (AI), soil nitrogen content (soil N) and soil C:N account for the variation in SLA along the transect? (ii) How does SLA of the dominant genera change with MAP, MAT, AI, soil N and soil C:N? (iii) What is the relationship between SLA and *N*_mass_ along the transect, and is such relationship subject to precipitation level?

**Table 1:** Basic information of the study sites along the transect.

Study sites	Latitude (N)	Longitude (E)	Elevation (m)	MAP (mm)	MAT (°C)	Genus^†^
1	40.88	104.45	1455	93	6.13	*St*
2	40.73	105.61	1293	99	6.93	*St*
3	41.45	107.00	1613	142	4.46	*St*
4	41.80	107.47	1512	151	4.28	*St*
5	41.83	107.61	1517	156	4.11	*St*
6	41.87	108.05	1384	173	4.47	*St, Cl*
7	42.16	109.17	1279	189	4.59	*St, Cl*
8	42.42	109.81	1151	180	5.33	*St*
9	42.62	110.29	1125	170	5.29	*St, Cl, Ca*
10	42.93	110.82	1035	152	4.92	*St, Cl, Ca*
11	43.15	111.36	1047	148	4.27	*St, Cl, Ca*
12	43.38	111.96	1015	148	3.56	*St, Cl*
13	43.63	112.20	959	147	3.50	*St, Cl, Ca*
14	43.71	112.92	1050	180	2.94	*St*
15	43.82	113.47	1022	199	2.72	*St, Cl, Ca*
16	43.85	114.09	1045	222	2.16	*St, Cl, Ca*
17	43.98	114.83	1126	246	1.13	*St, Cl, Le, Ca*
18	43.93	115.70	1089	271	1.32	*St, Le, Ca*
19	44.22	116.51	1095	305	1.02	*St, Cl, Le, Ca*
20	44.47	117.18	1049	324	1.03	*St, Cl, Le*
21	44.67	117.90	1111	360	0.51	*St, Cl, Le*
22	44.99	118.75	986	380	0.89	*St, Cl, Le, Ca*
23	45.43	119.72	969	420	0.42	*Le*
24	46.38	120.48	675	436	0.20	*Cl*
25	47.66	119.30	867	352	−1.85	*St, Le*
26	48.09	118.46	716	285	−1.04	*St, Cl, Le*
27	48.34	117.98	573	270	−0.26	*St, Cl, Le*
28	48.50	117.15	589	260	0.18	*St, Cl, Le*
29	48.86	116.89	568	262	0.32	*St, Le*
30	49.34	117.09	721	297	−1.49	*St, Cl, Le*
31	49.53	118.01	582	318	−1.48	*Cl, Le*
32	49.78	118.53	535	332	−1.47	*St, Cl, Le*
33	50.05	119.28	530	362	−1.71	*Cl*
34	49.88	119.99	762	360	−1.28	*St, Le*
35	49.48	119.68	599	360	−1.28	*Le*
36	49.19	120.36	633	392	−1.29	*Le*
37	44.77	123.38	142	421	5.32	*Le*
38	44.48	123.48	147	434	5.37	*Le*

^†^ *St* = *Stipa* spp., *Cl* = *Cleistogenes* spp., *Le* = *Leymus chinensis*, *Ca* = *Carex* spp.

**Table 2:** Main species and the distribution of all four genera mentioned in this study.

Genus	Main species	Distribution
*Stipa*	*Stipa krylovii*	From northwest to northeast in China
	*Stipa grandis*	
	*Stipa baicalensis*	
*Cleistogenes*	*Cleistogenes chinensis*	Inner Mongolia, Heilongjiang, Jilin provinces in China
	*Cleistogenes squarrosa*	
*Leymus*	*Leymus chinensis*	Eastern Inner Mongolia, western to northeast in China
*Carex*	*Carex pediformis*	Northeast, northwest, north and southwest mountain area in China
	*Carex korshinskyi*	

## Results

### Relative importance of climate and soil factors accounting for SLA variation

Multivariate analyses declared that the four genera have very different models to predict SLA variation. For *Stipa*, the best model included MAP, MAT, soil N, soil C:N and the interactions of soil N with MAP and with MAT, which together explained 71.65% of SLA variation. For *Cleistogenes*, the best model included MAT, soil N and their interaction, together accounting for 56.64% of SLA variation. For *Leymus*, the best model included MAT and soil N, accounting for 7.14% and 38.11% of SLA variation respectively. For *Carex*, the best model included MAP, MAT, soil N, soil C:N and the interaction between soil N and MAT, which explained 85.65% of SLA variation (Table 3).

**Table 3:** Multiple mixed regression relationships between SLA of the four genera and environmental variables.

Genera	Best models	n	r^2^	AIC	Explains %					
					MAP	MAT	Soil N	Soil C:N	MAP ~ Soil N	MAT ~ Soil N
*Stipa*	MAP + MAT + Soil N + Soil C:N + 2 interactions	30	0.717***	24.289	24.644	12.113	7.556	15.611	8.617	3.109
*Cleistogenes*	MAT + Soil N + 1 interaction	22	0.566**	56.872	—	3.700	12.463	—	—	40.473
*Leymus*	MAT + Soil N	20	0.453**	1.670	—	7.135	38.113	—	—	—
*Carex*	MAP + MAT + Soil N + Soil C:N + 1 interaction	10	0.857†	14.243	18.162	42.608	14.194	4.657	—	6.033

^†^0.05 < *P* < 0.1; *P < 0.05; **P < 0.01; ***P < 0.001.

### Bivariate relationships of SLA versus climate and soil factors

**Figure 2:**
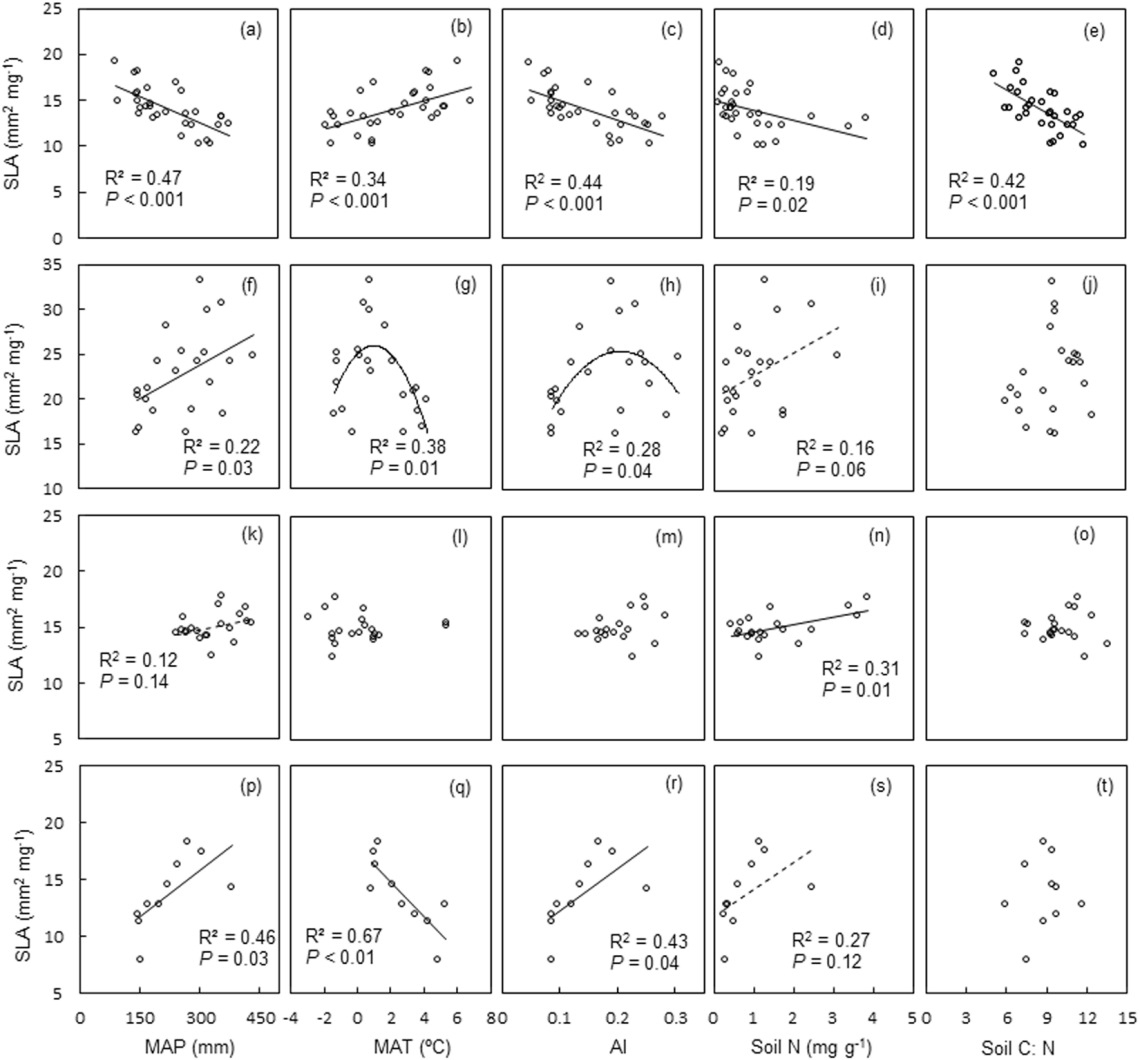
The statistics from regressions of SLA against MAP, MAT, AI, soil N and soil C:N in the whole transect. Panels (**a–e**) are for *Stipa*; panels (**f–j**) are for *Cleistogenes*; panels (**k–o**) are for *Leymus*; panels (**p–t**) are for *Carex*.

On the entire transect, the SLA vs. MAP and SLA vs. AI relationships were positive in *Carex*, not significant in *Leymus*, while negative in *Stipa* (Fig. 2). For *Cleistogenes*, the SLA vs. MAP relationship was positive while the SLA first increased and then decreased with AI. With increasing MAT, the SLA of *Cleistogenes* first increased and then decreased while the SLA vs. MAT relationship was negative in *Carex* but positive in *Stipa*. For *Leymus*, however, SLA was not significantly correlated with MAT (Fig. 2)*. Stipa* decreased and *Leymus* increased in SLA with the increase of soil N, while SLA of *Cleistogenes* and *Carex* showed increasing trends with soil N (Fig. 2). With increasing soil C:N, *Stipa* decreased in SLA while the other three genera were not significantly correlated (Fig. 2).

### Relationship between SLA and aboveground biomass

**Figure 3:**
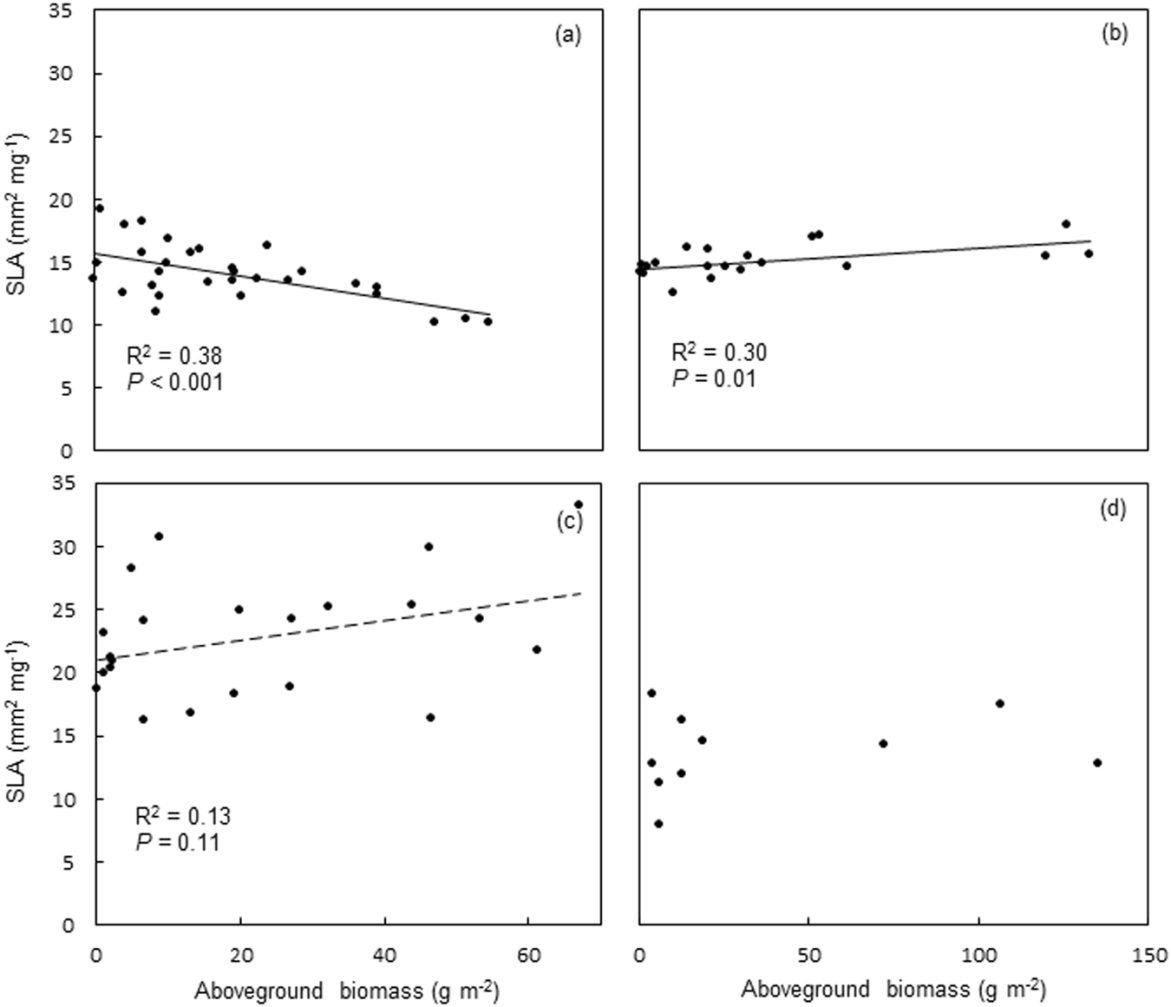
Relationships between SLA and aboveground biomass in each genus. Panel a is for *Stipa*; panel b is for *Leymus*; panel c is for *Cleistogenes*; panel d is for *Carex*.

The SLA and aboveground biomass were negatively correlated in *Stipa* (Fig. 3a), but positively correlated in *Leymus* (Fig. 3b). There is a trend that SLA of *Cleistogenes* increases with the aboveground biomass of the genera in the community, but statistically, such trend is only close to marginal significant (Fig. 3c). For *Carex*, probably due to the low number of the observations for the genera, the SLA is not significantly correlated at all with the aboveground biomass of the genera in the community (Fig. 3d).

### Correlations between SLA and *N*_mass_

**Figure 4:**
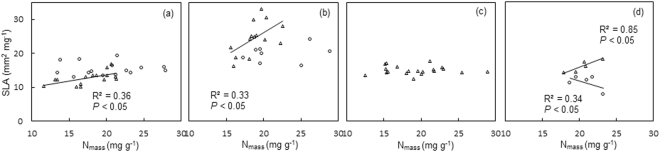
The relationship between SLA and *N*_mass_ for the regions with MAP < 200 mm (open circle) and MAP > 200 mm (open triangle). Panel a is for *Stipa*; panel b is for *Cleistogenes*; panel c is for *Leymus*; panel d is for *Carex*.

On the entire transect, SLA was positively correlated (*P* < 0.05) with *N*_mass_ in *Stipa*, but such correlation was not significant in the other three genera (Fig. 4). In the arid region where MAP was below 200 mm, SLA was negatively correlated with *N*_mass_ in *Carex* (Fig. 4d), although such correlation was not true in *Stipa* and *Cleistogenes* (Fig. 4a and b). In the semi-arid region where MAP was above 200 mm, however, SLA vs. *N*_mass_ relationship was significantly positive in *Stipa*, *Cleistogenes* and *Carex*, but not significant in *Leymus* (Fig. 4c).

## Discussion

Basically, our results about the relationship between SLA and plant aboveground biomass corroborates the notion that SLA is a proxy of the growth and abundance of the plants in the communities, which has been claimed in numerous published papers^6–8, 26, 28, 29^. This validates all the studies, including ours, that explicitly or implicitly used SLA as the proxy of plant growth. Our results declared that environmental variables have different impacts on SLA among different genera. This indicated that primary environmental factors determining SLA were genus-specific and just as Ordoñez *et al*. claimed, not all environmental variables were equally good predictors of SLA^7^. Between-genera discrepancy in the responses of SLA to environmental factors might be attributed to the fact that they belong to different functional groups and/or that they are different in anatomy and chemical composition^6^. Overall, climate variables play more important roles than soil fertility in SLA variation along this transect, probably because our study focused on a single biome, i.e., grassland, where the plant growth is primarily controlled by hydrothermal factors. In contrast, the relationship between SLA and *N*_mass_ are much more consistent, which reflects a trade-off between leaf strategies of fast growth vs. leaf longevity, corresponding to rapid acquisition vs. conservation of soil water and nutrients respectively^6, 7, 26^.

### Effects of climatic factors

Both multivariate and bivariate analysis indicated that climatic factors are more important than the investigated soil properties in determining SLA, which contrasted with Ordoñez *et al*.^7^. For instance, multivariate analysis showed that MAP and MAT account much more for SLA variation than soil nutrients in *Stipa* and *Carex*. The positive relationship between SLA and MAP in *Cleistogenes*, *Leymus* and *Carex* along the transect was consistent with Wright *et al*.^2^. Several other studies also found that plants from drier sites had lower SLA^23, 30^, which was interpreted as a mechanism whereby plants adapted to arid environment by keeping smaller but thicker leaves to decrease water loss. While plants growing in water- and/or nutrient-rich environments are able to obtain sufficient water and/or nutrients for fast growth, leaves become thinner and larger to promote their light capture in order to enhance their competitive capacities^30^.

SLA was negatively correlated with MAP and with AI in *Stipa*, which was really beyond our expectation, but some other inconsistent results had also been reported in *Stipa* plants before. Hu *et al*. found that SLA of *Stipa purpurea* varied little with mean growing season precipitation in alpine arid and semi-arid grasslands in Tibetan Plateau^31^. Tian *et al*. reported that SLA of *Stipa krylovii* was lowest under normal rainfall, and became greater whether the rainfall increased or decreased in typical steppe in Inner Mongolia^32^. In our study, both the leaf area and leaf dry mass of *Stipa* increased with increasing MAP, whereas the SLA decreased along the transect. We suspect that the unconventional responses of SLA of *Stipa* to precipitation might be attributed to the unique morphology or anatomy of *Stipa* leaves.

As perennial xerophytic herbaceous plant, *Stipa* adapts to drought environment by rolling up its leaves towards needle shaped, and this contributes to its dominance in the steppe communities. Moreover, the cross section of *Stipa* leaves completely curled up into a “V” or oval shapes to lessen the leaf area directly exposed to the air. Meanwhile, the outer surface of *Stipa* leaves is covered by a thick cuticle to increase the moisture diffusion resistance, leading to higher water use efficiency of leaf mesophyll cells^31, 33^. These mechanisms could contribute to *Stipa*’s dominance in the steppe communities by strong drought resistance (Table 2).

Correlations between SLA and MAT were quite inconsistent among the investigated plant genera in our study. Previous studies have drawn contrasting conclusions on the relationship between SLA and air temperature^2, 6, 7^. For instance, Wright *et al*. found that SLA was negatively correlated with MAT at global scale while Poorter *et al*. indicated that SLA increases with temperature^2, 6^. However, Ordoñez *et al*. found that there was no significant correlation between SLA and MAT^7^. Recently, De Frenne *et al*. did a meta-analysis and found that positive, negative and non-significant patterns in SLA vs. temperature relationship were all identified in previous studies^34^. Atkin *et al*. also claimed that the impact of air temperature on SLA was species-specific^22^. This suggests that different species have different mechanisms to cope with temperature changes, because distinct resource requirements across plant species can lead to different responses of species to the same environmental change^35^. It is possible that different responses of SLA to temperature are caused by plants’ fundamental growth characteristics and specific ecological niche^36^. Probably, these genus-specific responses are the pre-requisition and also consequences of an evolutionary process where different species fulfill complementary roles during community assembly (the niche complementarity hypothesis)^6, 37^.

### Effects of soil N and soil C:N

Taken together the results of multivariate and bivariate regressions, soil N, as the most commonly used proxy of N supply, accounts much more than soil C:N in explaining SLA variation along the transect. Although Ordoñez *et al*. deemed soil N as a rough estimate of nutrient supply^7^, we showed that high plant SLA were found at high soil N region, indicating that soil N was a decent predictor of SLA variation in temperate grassland, especially for nitrophilous *Stipa* and *Leymus* ^38^. Since SLA reflects plants’ abilities to access resources, our results implied that plants allow fast use of nutrients and rapid growth in fertile habitats, while small SLA occurred at low nutrient supply areas where conservation of nutrients is more important. With the increase of soil N, plants allow to obtain more nutrients to increase leaf area and further increase the photosynthetic capacity. This was in line with previous studies^4, 7^. Although Soil C:N can provide more information about the potential to mineralize or immobilize nutrients, it turns out to be a weaker predictor for SLA variation than soil N in temperate grassland, in contrast with the global synthesis incorporating various biomes^7^. Probably, this should be attributed to the nice linear relationship between soil C and N content in the temperate grassland, giving rise to a relatively very narrow range of C:N values.

The SLA vs. soil factor relationship was most distinctive in *Stipa* in our study. The variation of this association coincides with the SLA vs. MAP relationship in *Stipa*. With opportunistic growth patterns^39^, *Stipa* own distinctive morphological features and traits to deal with extreme environmental stress and allow its dominance in dry areas^40^. As soil N was positively correlated with humidity, interspecific competition become more intense with increasing MAP and soil N, and *Stipa* may keep longer, heavier leaves of smaller SLA to retain its competitive advantages^39^.

### Trade-offs between SLA and *N*_mass_

The relationship between SLA and *N*_mass_ was found to be dependent on precipitation levels. When MAP > 200mm, SLA was positively correlated with *N*_mass_, in line with previous studies from regional to global scale with MAP ranging from 133 to 5,300 mm^1, 2, 19, 24, 25^; while in the region of MAP < 200 mm, SLA was unrelated or negatively correlated with *N*_mass_. It has been claimed that the correlation between SLA and *N*_mass_ reflects the trade-off between two opposing resource strategies, i.e., conservation vs. rapid acquisition of soil water and nutrients^2, 26, 41^. In those regions that receive plentiful rainfall, plants obtain sufficient nutrients for fast growth in order to enhance their competitive capacities, implying that high SLA and *N*_mass_ reflect the plant strategy of rapid resource acquisition. However, in the regions where MAP < 200 mm, both nutrients and water are in shortage, plants have smaller SLA to reduce the transpiration^6^, while *N*_mass_ were increased to improve cellular osmotic pressure and strengthen water protection in plants, in order to resist drought stress^42, 43^. Therefore, low SLA and high *N*_mass_ reflect the resisting drought stress strategy of plants. Tian *et al*. investigated the responses of leaf traits of 14 plant species in the typical steppe to simulated rainfall, but failed to find simple linear relationship between SLA and rainfall, instead they found that *N*_mass_ was affected by simulated rainfall^32^. This result might explain why SLA vs. *N*_mass_ relationship in our case was contrasting among genera and was subject to MAP level.

## Conclusions

Summarily, climatic factors account much more for SLA variation than soil variables for *Stipa*, *Cleistogens* and *Carex*. SLA of *Stipa* is negatively associated with MAP, AI and soil N, while positively with MAT, and *Cleistogens* and *Carex* show the opposite. For *Leymus*, SLA variation can be explained primarily by soil N, with a positive correlation. Such genus-dependent responses of SLA may have implications in terms of plant coexistence under the future climate scenario, as different species fulfill complementary roles during community assembly. In addition, since SLA is a key determinant of plant growth and playing an important role in plant community assembly^2, 6^, it is bound to have consequences on ecosystem functioning. For example, high SLA plants are preferentially attacked by herbivores, and also decompose much faster, facilitating carbon and nutrient cycling, which in turn enhances the primary production^6^. Therefore, our results may help to predict the changes of community productivity, species distribution range, and other ecosystem functions under the context of climate change. Based on our results, we predict that with the aridifying and warming climate, the productivity of the four dominant genera will decrease and their distribution range will shift northeastward in China. Moreover, as SLA is a key functional trait reflecting the trade-off between resource capture and conservation^2, 6^, our findings on the SLA-*N*_mass_ relationship and its susceptibility to precipitation are bound to advance our understanding on plant adaptive strategies.

## Materials and Methods

### Study area

The investigation was conducted along an essentially west-east transect of approximately 2500 km long in temperate grassland in Northern China, from Alxa Left Banner, Inner Mongolia in the west to Changling County, Jilin Province in the east, ranging from 40.7°N to 50.1°N in latitude and 104.5°E to 123.5°E in longitude (Fig. 1; Table 1). The range of elevation was between 142 m to 1613 m a.s.l. The range of soil water content was from 2% to 28% and nutrient availability gradually increased from west to east along the transect^44^. The vegetation types along the transect included desert steppe, typical steppe and meadow steppe from west to east according to *Vegetation of China* ^45^. Three climatic variables AI (calculated as the ratio of precipitation to potential evapotranspiration), MAT and MAP were examined in this study, to analyze the effects of climate on the changes in SLA. MAP ranges from 93 to 436 mm and MAT ranges from −1.85 to 6.93 °C along the climatic gradient. The data of MAT and MAP were extracted from the WorldClimdatabase^46^ from 1950 to 2000.

### Sampling

The four most widely distributed dominant genera in the temperate grasslands of northern China and adjacent Mongolia, *Stipa*, *Cleistogenes*, *Leymus* and *Carex* were selected as the plants of interest (Table 2). Sampling was carried out during the first half of August in 2012, a period during which the phenological changes in these plants were relatively negligible and plants were fully mature throughout the transect. We selected 38 sample sites along the transect (Fig. 1; Table 1). All the sampling sites were selected inside the enclosures, which were very common in China grassland region due to grazing ban policy. So our study sites had been subject to minimal grazing and other anthropogenic disturbances. Each site was geo-referenced with GPS (eTrex Venture, Garmin, ±3 m accuracy) for its latitude, longitude and elevation. At each site, five quadrats (1 m × 1 m each) were selected for both vegetation survey and soil sampling. In each quadrat, after removing floor litter, 20 random soil samples (0–10 cm) were collected using a soil core (2.5 cm diameter), and were mixed together into one sample. Soil samples were air-dried right after sampling and then stored in a plastic bag for measurements. Plant samples were collected randomly next to the five quadrats at each site. Where present, five to ten adult and healthy individuals without obvious symptoms of pathology or herbivore attack were randomly sampled for each of the four genera^11^. We collected the whole aboveground parts and put them in sealed plastic bags, storing them in a refrigerator at 4 °C until further processing in the laboratory later the same day.

### Measurements

In the laboratory at least 20 young but fully expanded and hardened leaves were taken from the 5 to 10 randomly-selected individual plants for each of the four genera at each site. Each leaf was cut off from the culm and gently rubbed dry before measurement^11^. We used a scanner (HP Scanjet G3110) to scan each leaf image; then leaf area of each leaf was calculated using software Image J^47^. For very small or very narrow leaves like *Stipa*, transparent scotch tape was used to fix them onto blank paper before scanning. Each leaf sample was then dried at 80 °C for 48 h and weighed.

Soil samples were passed through a 2 mm mesh sieve to remove fine roots and plant debris in laboratory, then the samples were homogenized by hand mixing and oven-dried at 65 °C to constant weight. Both plant and soil samples were ground in a ball mill (NM200, Retsch, Haan, Germany) and then stored in a plastic bag until further analysis. Leaf and soil total N concentration (expressed here per unit of dry mass, mg·g^−1^) and C concentration (per unit of dry mass, mg · g^−1^) were determined for each sample with an elemental analyzer (Vario EL III, Germany). Since all the plant and soil samples were collected by a single team of researchers using uniform sampling method within a period of 15 days under constant weather conditions, our analysis avoids the difficulty of heterogeneous data as encountered in previous large-scale surveys^48^ or synthesis studies^7, 8^.

### Statistical analysis

Levene’s test was used to test for normality of all data before statistical analysis. Bivariate analysis and multivariate analysis were performed to relate SLA and environmental variables (MAP, MAT, AI, soil C:N, soil N) separately for each genera. When analyzing the SLA vs. *N*_mass_ tradeoff, we divided the total sites pool into two parts: semi-arid areas (sites in the area with >200 mm MAP) and arid areas (sites in the area with <200 mm MAP) according to *Climate classification in China* ^49^. Relationships between SLA and *N*_mass_ in the two different MAP parts were analyzed using linear regression. All the above statistical analyses except multivariate analysis were performed with SPSS 18.0 (SPSS Inc., Chicago, IL, USA) for windows. Multivariate analysis of the effects of soils and climate on SLA was performed using the R software package (version 3.2.0) and the Akaike information criterion (AIC) was selected as a goodness of fit measure: the lower the AIC value, the better the model.
